# Ultrafast x-ray diffraction study of melt-front dynamics in polycrystalline thin films

**DOI:** 10.1126/sciadv.aax2445

**Published:** 2020-01-17

**Authors:** Tadesse A. Assefa, Yue Cao, Soham Banerjee, Sungwon Kim, Dongjin Kim, Heemin Lee, Sunam Kim, Jae Hyuk Lee, Sang-Youn Park, Intae Eom, Jaeku Park, Daewoog Nam, Sangsoo Kim, Sae Hwan Chun, Hyojung Hyun, Kyung sook Kim, Pavol Juhas, Emil S. Bozin, Ming Lu, Changyong Song, Hyunjung Kim, Simon J. L. Billinge, Ian K. Robinson

**Affiliations:** 1 Condensed Matter Physics and Materials Science Department, Brookhaven National Laboratory, Upton, NY 11793, USA.; 2 Department of Applied Physics and Applied Mathematics, Columbia University, New York, NY 10027, USA.; 3 Department of Physics, Sogang University, Seoul 04107, Korea.; 4 Department of Physics and POSTECH Photon Science Center, Pohang University of Science and Technology, Pohang 37673, Korea.; 5 Pohang Accelerator Laboratory, Pohang, Gyeongbuk 37673, Korea.; 6 Computational Science Initiative, Brookhaven National Laboratory, Upton, NY 11793, USA.; 7 Center for Functional Nanomaterials, Brookhaven National Laboratory, Upton, NY 11793, USA.; 8 London Centre for Nanotechnology, University College London, London WC1E 6BT, UK.

## Abstract

Melting is a fundamental process of matter that is still not fully understood at the microscopic level. Here, we use time-resolved x-ray diffraction to examine the ultrafast melting of polycrystalline gold thin films using an optical laser pump followed by a delayed hard x-ray probe pulse. We observe the formation of an intermediate new diffraction peak, which we attribute to material trapped between the solid and melted states, that forms 50 ps after laser excitation and persists beyond 500 ps. The peak width grows rapidly for 50 ps and then narrows distinctly at longer time scales. We attribute this to a melting band originating from the grain boundaries and propagating into the grains. Our observation of this intermediate state has implications for the use of ultrafast lasers for ablation during pulsed laser deposition.

## Teaser

Laser melting of polycrystalline metals starts at the internal grain boundaries and propagates into the grains.

## INTRODUCTION

Understanding the role and behavior of transient states during phase transitions, such as melting, is becoming increasingly important in condensed matter physics (*1*–*4*). Time-resolved (TR) pump-probe techniques are well suited to capture these transient states. An optical laser pump pulse induces the phase transition, and an ultrashort x-ray/electron pulse analyzes the properties of the transient state, which follows after a specified time delay. Pump-probe methods have been used to study time evolution (*5*, *6*) of thermal and nonthermal melting processes in metals (*7*–*9*) and semiconductors (*10*, *11*). These and other experiments have been widely interpreted with the help of the two-temperature model (TTM) (*12*), in which the pump pulse is considered to create hot electrons that subsequently transfer their heat to the crystal lattice through electron-phonon coupling within a few picoseconds (*13*). This model is generally accepted as describing the laser excitation process in a wide range of materials and has been incorporated into a TTM molecular dynamics simulation method, which has led to simulations that are found to be in good agreement with experimental results (*14*–*16*).

Macroscopic materials are often polycrystalline, containing a large density of differently oriented grains separated by grain boundaries (GBs). Melting has been widely studied over many decades and is believed to be initiated preferentially at defects, such as surfaces, dislocations, GBs, stacking faults, and point defects that contain atoms with lower coordination number, thus more dangling bonds than in the bulk (*17*, *18*). According to the Lindemann criterion, which is when the amplitude of atomic vibration reaches 10% of the nearest neighbor atomic distance, melting happens at lower temperatures near these defects. Experiments, for example, by ion channeling, have shown an increase in disorder at these locations slightly below the bulk melting temperature (*19*). Theoretical studies have confirmed this concept that melting in granular materials starts to nucleate at dislocations and GBs (*20*, *21*). While the location of the initiation of melting at these weak points of polycrystalline material is widely accepted, the time dependence of the progression of melting is an open question, which has been much less studied. Ultrafast electron diffraction experiments have explored the time scales of melting in free-standing single-crystal films and found different melting regimes (*7*).

Furthermore, it is well known that the presence of GBs affects electron transport in metals, most easily seen in their electrical conductivity (*22*). In metal thin films, the electron-phonon coupling constant is related to the inelastic mean free path (λ) of electrons, which varies with energy according to the “universal curve” (*23*). In addition, it depends on the electron reflection coefficient from defects such as GBs, film thickness, and grain size (*24*, *25*). Depending on the electron energy, the inelastic mean free path is in the range of a hundred nanometers. This implies that most of the electrons generated from the front side of a 300-nm sample do not make it to the other side, which agrees with experiments (*13*). In a polycrystalline thin film, however, the separation between GBs, often assumed to scale with the film thickness, can dominate once again and provides a characteristic thickness dependence of resistivity (*22*). Within the framework of the TTM, it is expected that the hot electrons will couple to the lattice preferentially at GBs due to this additional electron scattering (*26*, *27*). Electrical transport measurements indicate slower effective electron velocities in polycrystalline than in single-crystal gold thin films, due to the additional scattering at GBs (*28*). This results in a characteristic penetration depth of ultrafast hot electrons, which is considerably longer than the 12-nm electromagnetic skin depth at 400-nm laser wavelength in polycrystalline gold films (*13*). For both these reasons, we would expect to see heterogeneous melting in a granular metal when it is excited by a laser, with preferential melting at the GBs.

Our experiment was therefore designed to explore the time dependence of laser-induced structural changes in polycrystalline gold (Au) thin films of 50-, 100-, and 300-nm thicknesses prepared with the electron beam evaporation technique. A femtosecond laser pulse was used to excite electrons at the front surface of the Au thin film with a range of fluences spanning the level needed to fully melt the film in a single shot. By measuring the x-ray diffraction (XRD) patterns of the film with a single x-ray pulse produced by the Pohang Accelerator Laboratory X-ray Free Electron Laser Facility (PAL-XFEL), we monitored the structural changes following a 400-nm optical laser excitation.

## RESULTS

### Single-shot TR XRD results

**Fig. 1:**
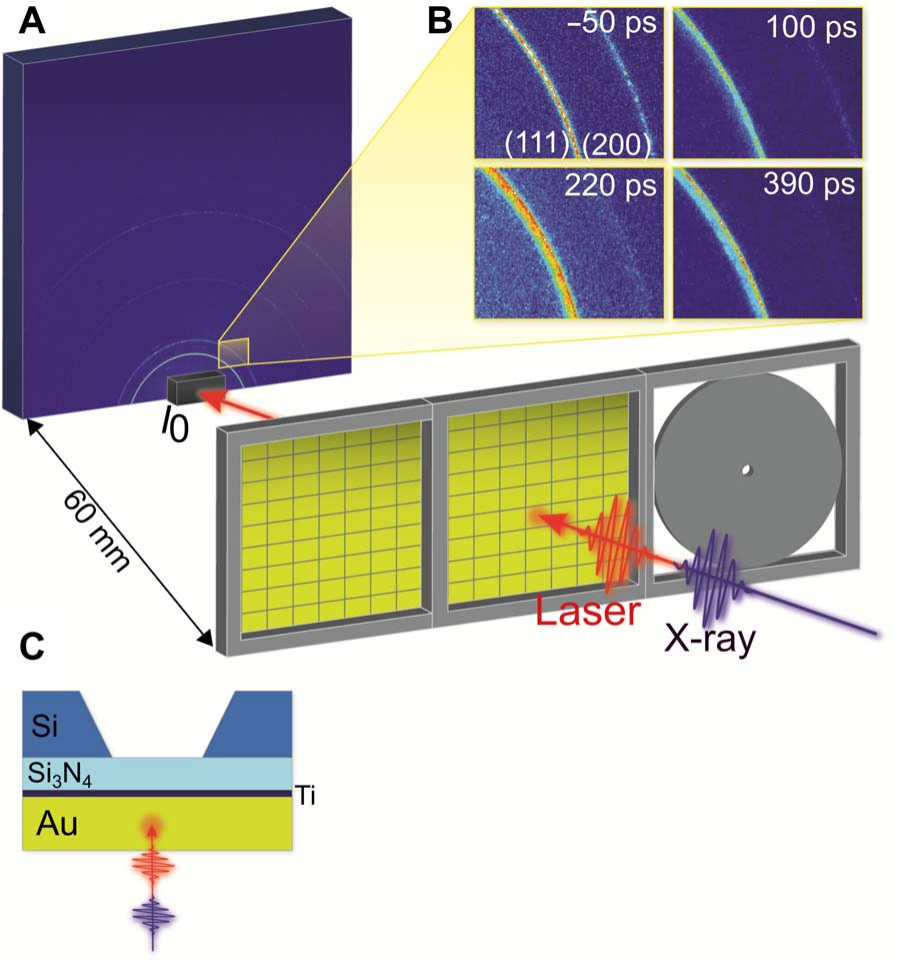
Experimental configuration for TR XRD from polycrystalline gold thin films. (**A**) Experimental setup showing the sample, the Rayonix detector MX225-HS, and the photodiode (*I*_0_) to measure the transmitted beam for normalization. The sample was mounted perpendicular to the 9.7-keV x-ray beam, and a 400-nm, 100-fs optical laser was used to excite the sample in almost colinear geometry. (**B**) 2D diffraction patterns of the 300-nm thin film collected 50 ps before and 100, 220, and 390 ps after laser excitation at an incident laser fluence of 254 mJ/cm^2^. (**C**) Cross-sectional view of the gold thin film and substrate window array arrangement.

**Fig. 2:**
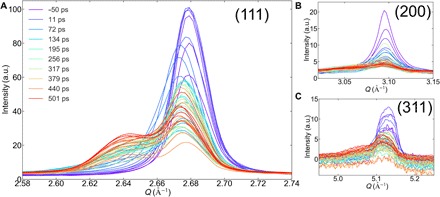
Dynamics of azimuthally integrated XRD profiles of different peaks. (**A**) (111) (**B**) (200), and (**C**) (311) diffraction peaks for the 300-nm-thick thin film measured at various delay times after an incident laser fluence excitation of 254 mJ/cm^2^. All the diffraction positions have both the crystal powder and intermediate peaks. a.u., arbitrary units.

The XRD data were collected in a transmission Debye-Scherrer geometry to minimize background shown in Fig. 1A. A new broader “intermediate” peak is seen to appear on the lower *Q* side of the (111) powder ring shown in Fig. 1B. The new powder ring observed here has less azimuthal intensity variations than the (111) powder ring, indicating that the contributing material is more homogeneous. To show the full *Q* dependence, we azimuthally integrated each measured two-dimensional (2D) image to give the XRD profile curves shown in Fig. 2 (A to C). The powder (111), (200), and (311) Bragg peaks all show two clear peaks, a sharp powder peak, and a broader intermediate diffraction peak at lower *Q*, which becomes visible about 50 ps after laser excitation.

**Fig. 3:**
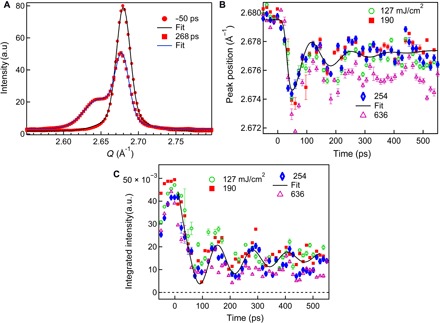
Dynamics of the (111) crystal diffraction peak measured from 300-nm gold thin films. (**A**) Fits to the diffraction intensity profiles versus momentum transfer, *Q*, measured 50 ps before and 268 ps after excitation at an incident laser fluence of 254 mJ/cm^2^. (**B**) Peak position and (**C**) integrated intensity as a function of time delay. For the 254 mJ/cm^2^ data, the peak position was fitted with a sum of exponential decay (time constant, τ_1_ = 2800 ± 50 ps) and an exponentially damped cosine function (with damping time constant, τ_2_ = 90 ± 10 ps, and period, *T* = 130 ± 10 ps). Similarly, an integrated intensity of 254 mJ/cm^2^ was fitted with τ_1_ = 2800 ± 100 ps, τ_2_ = 250 ± 45 ps, and *T* = 130 ± 10 ps.

To understand the temporal evolution of the laser-induced changes in the sample, we fitted the XRD profiles measured at different times with two components, as shown in Fig. 3A, with full details in Materials and Methods. Both the crystal peak position and intensity, shown in Fig. 3 (B and C), are seen to decay and oscillate; the oscillation strength depends weakly on the incident laser fluence. The oscillatory behavior has been explained before to originate from longitudinal acoustic vibration waves traversing the film with a period proportional to the sample thickness (*29*, *30*), while the decay is explained by partial melting (*3*, *7*).

### Origin of the intermediate peak

The intermediate peak position, width, and integrated intensity are well described by the Gaussian function used in the fitting procedure (details below), as seen in Fig. 3A. While the intermediate peak position, around 2.655 Å^−1^, is close to the first peak of liquid gold at 2.64 Å^−1^ (*31*), the peak is 10 times sharper than any known liquid gold peak, so it cannot be attributed to a conventional equilibrium liquid state. Analogous intermediate peaks are also observed near the (200) and (311) powder peaks (in Fig. 2, B and C), which do not exist at all in the liquid structure factor (*32*). The peak also cannot be due to the effect of thermal expansion with a continuous distribution of crystal temperatures within the sample because that would give only a monotonic tail on the crystal diffraction peak. Instead, the formation of a distinct peak due to thermal expansion implies that there has to be part of the material with a well-defined larger lattice parameter, hence preferred temperature. The only singular temperature of gold is the melting point itself, *T* = *T*_m_ (1338 K): The crystal temperature could be trapped there during heating, while it takes up the latent heat of melting. The crystal, undergoing melting, apparently retains a single, well-defined larger lattice constant while it becomes progressively disordered. The splitting of the peak into two distinct components, identified with two different temperatures, is a direct signature of inhomogeneous melting.

**Fig. 4:**
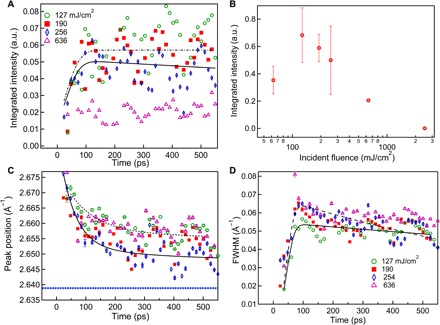
Dynamics of the intermediate diffraction peak of the (111) profile. (**A**) Integrated intensity, (**B**) integrated intensity of the intermediate peak at different incident fluences, (**C**) position, and (**D**) width as a function of pump-probe delay time for 300-nm films. The data measured at different incident laser fluences are indicated with different colors. The peak width was fitted with an exponential decay convoluted with a Gaussian function. For the data measured at 254 and 127 mJ/cm^2^, the time constants were 1320 ± 50 ps and 3900 ± 600 ps, respectively. The peak position measured at 254 mJ/cm^2^ was fitted to a sum of two exponential functions with time constants of 50 ± 10 ps and 330 ± 20 ps convoluted with a Gaussian function. The dashed blue line shown in (C) is the expected position of the gold (111) peak at the melting point due to thermal expansion, assuming ambient pressure.

The intermediate peak position, shown as a function of time in Fig. 4C, does not reach the position expected for the lattice parameter of gold at the melting point, shown as a horizontal blue dashed line in Fig. 4C. We consider that the peak position is offset by the effects of pressure in the melting layer, estimated to be around 5 GPa from the bulk modulus and thermal expansion coefficient. The pressure (*P*) was estimated using *P* = *K*_0_Δ*V*/*V*, where *K*_0_ is the isothermal bulk modulus for gold (167 GPa) and Δ*V*/*V* = 3(Δ*Q*/*Q*) is the relative volume change determined by the peak shift, Δ*Q*. Such an induced pressure would be expected to dissipate acoustically, and we note the presence of acoustic oscillations in the peak position, which are particularly strong at a fluence of 254 mJ/cm^2^, which also gives the strongest intermediate peak.

### Dynamics of the crystal diffraction peak

For the 300-nm thin film, upon laser excitation, the (111) crystal peak shows an abrupt reduction of intensity for all laser fluences used in our measurements. In addition, the (111) crystal peak position shows a negative position shift due to thermal expansion of the gold. For the data measured at an incident laser fluence of 254 mJ/cm^2^, the maximum excursion of the peak is Δ*Q* = −0.0047 Å^−1^ at 60 ps after laser excitation, corresponding to a crystal temperature change of 400 K, estimated from the thermal expansion coefficient of gold, 14 × 10^−6^ at 300 K (*33*), and assuming no pressure contribution.

**Fig. 5:**
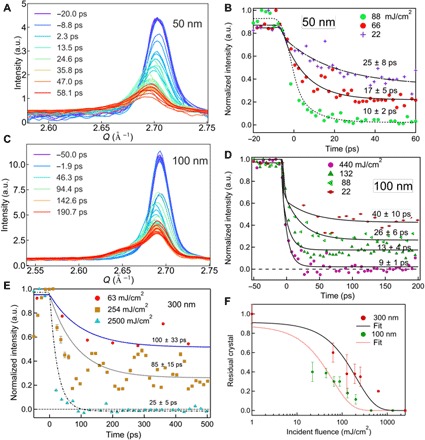
Thickness dependence of the melting time of gold thin films. (**A**) Diffraction intensity profiles measured from a 50-nm-thick gold film at an incident laser fluence of 50 mJ/cm^2^ at different delay times after laser excitation. (**B**) Time dependence of the integrated diffraction intensity of 50-nm thin films at different incident laser fluences. (**C**) Diffraction profiles of a 100-nm-thick gold film measured at an incident laser fluence of 66 mJ/cm^2^. (**D**) Integrated intensity of the (111) peak as a function of pump-probe delay time for different fluences. (**E**) Time dependence of the crystal peak component for the 300-nm gold thin films for different incident laser fluences. (**F**) Residual crystal fraction versus fluence for 100- and 300-nm-thick films. The fraction of the crystal peak intensity remaining 500 ps after laser excitation is plotted versus incident laser fluence and fitted with a sigmoid function.

In all the 300-, 100-, and 50-nm thin films, shown in Fig. 5 (B, D, and E), the sharp decrease in the (111) integrated peak intensity was fitted with an exponential decay function. The dynamics of the crystal peak integrated intensity is different for the different film thickness. For all the different film thicknesses reported here, the decay time was found to depend strongly on the incident laser fluence. The integrated intensities of the 300-nm film, measured at fluences of 63, 636, and 2500 mJ/cm^2^, were fitted with exponential decay times of 100 ± 33 ps, 85 ± 15 ps, and 25 ± 5 ps, respectively. These values are close to the observed rise times of the intermediate peak, consistent with a conversion of crystal into the material that gives rise to the new intermediate diffraction peak.

### Analysis of crystal peak oscillations

Both the integrated peak intensity and peak positions for the 300-nm films show both a decay and damped oscillations. This behavior is interpreted as due to loss of material to melting and acoustic wave oscillations. It is fitted with a function

A(t)=A0(1+A1e−tτ1)+A2(e−tτ2)cos(2πtT+ϕ)

where *A*_0_, *A*_1_, and *A*_2_ are amplitudes; τ_1_ and τ_2_ are decay times; *T* is the period of oscillation; and ϕ is a phase offset. Both τ_1_ and τ_2_ become shorter for higher fluence data; moreover, the time observed here is shorter than the decay time observed in gold nanocrystals (*1*), which were measured at fluences below the damage threshold. The damping time constant is close to the observed rise time of the intermediate peak, consistent with a conversion of crystal into the material giving the new intermediate diffraction peak. For the data measured at an incident laser fluence of 254 mJ/cm^2^, the fit to the peak position gives an oscillation period (*T*) of 136 ± 10 ps, corresponding to acoustic waves in a 270 ± 30–nm sample thickness propagating at a longitudinal speed of sound of 3690 m/s in gold films (*30*). A similar oscillation period was found to fit the integrated peak intensity.

### Temperature rise due to laser heating

The pulse energy (*E*_pulse_) was calculated by dividing the laser power by the 10-Hz repetition rate of the laser. The laser beam size, *d*, at full width at half maximum (FWHM) was estimated to be 100 μm at the sample position (full description is given in Materials and Methods). The incident laser fluence (*F*_in_) was then taken to be *E*_pulse_/*A*, where A=π(d24), *A* is the laser beam area. Assuming that the x-rays are probing the central part of the excited region of the sample, we estimate that a maximum temperature rise, Δ*T*, of the thin film of thickness *h* can be estimated as Δ*T* = (ϵ*F*_in_)/(*h*ρ*C*_P_), where ϵ = 0.6 is the absorption coefficient of gold thin at 400 nm (*7*), ρ is the density of gold (19.3 g/cm^3^), and *C*_P_ is the specific heat capacity of gold (0.128 J/g K). The absorbed fluence *F*_ab_ will be smaller than this reported value due to significant reflection from the surface of the sample. For experiments performed on the 300-nm film at incident laser fluences of 127, 190, 254, and 636 mJ/cm^2^, we estimated the temperature rises of 1030, 1538, 2060, and 5150 K, respectively, ignoring the latent heat contribution. These estimated temperature values are subject to uncertainties of the probe region. Whenever the laser heating goes into latent heat, *L* = 66 J/g, at the melting point *T*_m_= 1338 K, there will be a smaller temperature rise of 516 K. According to these estimates, an incident fluence of 254 mJ/cm^2^ should be just sufficient to melt the 300-nm film sample.

### Melting time and partial melting

From the estimates given above, we estimate that the absorbed fluence for complete melting should be 98, 33, and 16 mJ/cm^2^ for the 300-, 100-, and 50-nm thin films, respectively. A recent result (*7*) showed that the threshold for complete melting of 30-nm polycrystalline gold thin films was about 15 mJ/cm^2^, consistent with these numbers. In our data in Fig. 3C, for the 300-nm films, even an incident laser fluence of 636 mJ/cm^2^ is still not enough to completely melt the film because there is still some intensity left in the crystal powder peak. Both the crystal peak position in Fig. 3B and intensity in Fig. 3C show an exponential decay and oscillation in the remainder of the partially melted film. At higher fluences, both the peak position and intensity oscillations become weaker. The peak was found to disappear completely within 25 ps at an incident laser fluence of 2500 mJ/cm^2^, suggesting complete melting of the film at the level of sensitivity of our measurement.

We suppose that this incomplete melting, especially of thicker films, is explained by the limited transmission of the hot electrons through the sample, due to thermal scattering and scattering from the GBs. It was seen in the previous work (*13*) that only a small fraction of the hot electrons are able to traverse a 300-nm gold thin film. When the samples are driven with fluences well above the estimates above for complete melting, there must be a significant temperature gradient established by the limited electron transmission: While the far side of the sample is not yet reaching the melting point, the laser-illuminated side goes well above and may reach the boiling point and/or start ablating. This conclusion was tested by examination of the fluence dependence of the decay of the crystal peak intensity of 50- and 100-nm thin films in Fig. 5 (B and D). For the 100-nm thin film, we observe complete melting within 25 ps at an incident laser fluence of 440 mJ/cm^2^. Similarly, 20 ps is the melting time needed for the 50-nm film at an incident laser fluence of 88 mJ/cm^2^. Melting becomes faster as the incident laser fluence increases, in good agreement with the recently reported data on 35-nm polycrystalline thin films (*7*), which were interpreted as the sample crossing from heterogeneous to homogeneous melting. In Fig. 5F, we plot the residual fraction of crystal left after 500 ps of melting against the incident laser fluence, which shows a roughly sigmoid function with a threshold of 153 mJ/cm^2^ for the 300-nm film. The 100-nm thin film shows similar behavior with a threshold of 60 mJ/cm^2^.

## DISCUSSION

**Fig. 6:**
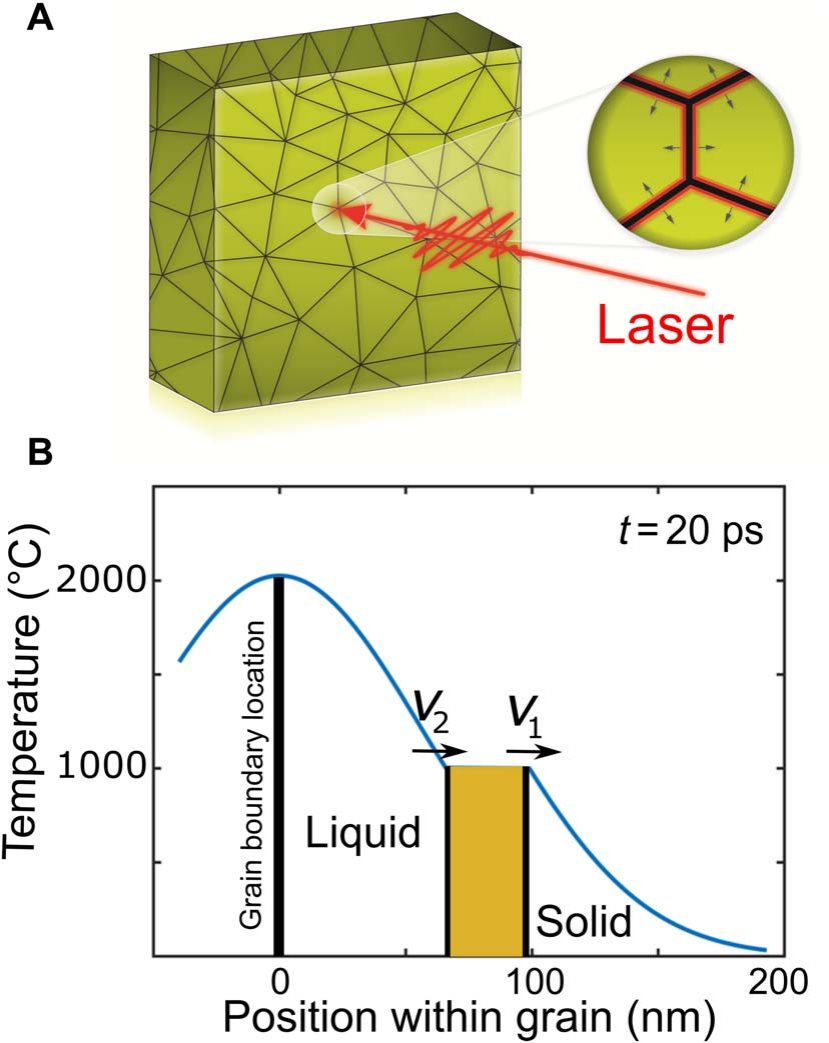
GB melting mechanism in a polycrystalline gold thin film. (**A**) Sketch of GB locations in a polycrystalline gold thin film and a zoomed view of how the melt front would propagate away from the GB following optical laser excitation. (**B**) Simulation of the spatial temperature distribution using the heat diffusion equation from a spike of melt created at the GB, at *x* = 0. The heat moves rapidly into the grain with a melt-front velocity determined by the heat flux. The shaded offset region is due to the uptake of latent heat, resulting in a block of melting material sandwiched between two melt fronts moving at different velocities.

Having identified that the intermediate peak arises from the material undergoing melting, we can then follow its behavior to report on the structure and properties of the melting region as a function of time and fluence. Since the peak is the structure factor of the melting material, we follow its trends, through line shape fitting, to understand the overall melting behavior. For the reasons given in Introduction, we assume that the melting initiates at the GBs of the polycrystalline film where the electrons couple preferentially to the lattice. Heat flow starting from the GBs causes a melting wave, which propagates toward the core of each grain as illustrated schematically in Fig. 6A.

### Melt-front velocity

Classical thermal diffusion calculations can be used to understand the propagation of melting, once the few-picosecond electron-lattice equilibration time of the TTM has elapsed. This is shown as a temperature-position profile in Fig. 6B, illustrating how the thermal spike assumed to start at the GBs diffuses rapidly into the neighboring grains and gives rise to a melt front where the temperature crosses the melting temperature, *T*_m_. The solution of the 1D heat diffusion equation starting from a point source at the GB where a pulse of heat is injected at time *t* = 0 is a spatial Gaussian distribution, *T*(*x*, *t*), of width $\sqrt{2\mathrm{kt}/C_{p}\rho }$, where *k* is the thermal conductivity, *C*_p_ is the specific heat, and ρ is the density. According to Fourier’s law, the heat flow, $\dot{Q}$ or (*dQ*/*dt*), is proportional to the spatial derivative of *T*(*x*, *t*), which is (*C*_p_ρ*A*/2)*T*(*x*, *t*) (*x*/*t*), where *A* is the area of the GB considered. When *T* = *T*_m_, the melting temperature, (*x*/*t*) can be considered as a melt-front velocity, *v*, determined by the rate of heat flow *v* =$2\dot{Q}/\rho AC_{p}T_{m}$. The heat flow, $\dot{Q}$, is given by the initial conditions and is expected to be proportional to the absorbed laser fluence, which explains why melting times become shorter with fluence, as we and others observe (*7*).

### Effect of latent heat

Because of the uptake of latent heat, there can be two melt fronts with different velocities *v*_1_ for the solid-melt boundary and *v*_2_ for the melt-liquid boundary, indicated in Fig. 6B. These are given by $\dot{Q}=(1/2)\rho Av_{1}(T_{m}C_{p})=(1/2)\rho Av_{2}(L+T_{m}C_{p})$, where *L* is the latent heat. This defines a band of melting material sandwiched between the two melt fronts with *T* = *T*_m_, which is responsible for the intermediate diffraction peak. Assuming a constant heat flow and that both fronts start together, they grow apart with a ratio, *v*_2_/*v*_1_ = (*L* + *T*_m_*C*_p_)/(*T*_m_*C*_p_) = 1.39, resulting in enlargement of the melt region.

### Explanation of narrowing of the intermediate peak

As the melting region propagates through the grain, the gold region undergoing melting between the two melt fronts becomes larger in time, according to this model. While the associated diffraction peak in Fig. 4 shows a rise in both its integrated intensity and width within the first 50 ps, the most significant trend is a distinct narrowing of the peak width over the 100- to 500-ps delay range in Fig. 4D. We interpret this to be due to the widening band between the two melt fronts. The physical size of the band, given by 2πW, where *W*, the FWHM of the intermediate peak, is 10 nm at 100 ps growing to 14 nm at 550 ps for 254 mJ/cm^2^. These values, together with the model above, provide an estimate of the melt-front velocity, *v* = 30 m/s. Only the integrated intensity shows significant fluence dependence, shown in Fig. 4A, with a drop of intensity for the higher fluence, attributed to faster melting. We interpret the initial 50-ps rise time as the time needed to establish the melt front, while the quasi-stable behavior following this transition corresponds to a steady progression of the melt front while energy is being transferred from the latent heat to the melt.

### Summary and implications

Our comprehensive picture of the ultrafast melting of polycrystalline gold films starts when the laser pulse is absorbed within the electromagnetic skin depth of the sample (12 nm at 400 nm wavelength), creating a population of hot electrons, which then travel through the sample at Fermi velocity (*12*, *34*). During this process, hot electrons transfer their energy to the lattice preferentially at the GBs (*27*), leading to inhomogeneous melting with a pair of melt fronts being emitted from each GB, as illustrated in Fig. 6. This melting material gives rise to a well-defined new diffraction peak with quasi-static width, position, and intensity living beyond 500 ps, suggesting that the thermally expanded crystal lattice is partially preserved at *T* = *T*_m_ during the transfer of its latent heat at the melting point. Polycrystalline thin films are prone to have inhomogeneities associated with their GBs, surfaces, dislocations, stacking faults, and point defects. All these inhomogeneities will have atoms with lower coordination number than in the bulk, leading to spatial inhomogeneities in the electron-phonon coupling rate. For metals, the electron-phonon coupling rate increases with the density of GBs (*22*, *23*). According to (*24*), increases in the number of GBs lead to a decrease in the mean free path of electrons due to an increase in electron scattering locations. When a femtosecond laser excites a polycrystalline metal thin film, the hot electrons generated will efficiently transfer energy to the lattice at these electron scattering locations. This allows more precise machining of materials with ultrafast lasers (*35*). It is found that granularity appears when nanosecond lasers are used, so femtosecond lasers are advantageous for precise micromachining, which significantly reduces the formation of a “heat-affected zone” in a material (*36*). Moreover, this will also have a consequence for laser ablation, perhaps contributing to the formation of particulates found in the ablated plume (*37*). In both applications, our model may explain why grain averaging when using nanosecond lasers might be needed.

## MATERIALS AND METHODS

### Thin film sample preparation

Gold films with a nominal thickness of 50, 100, and 300 nm were fabricated by electron beam evaporation onto standard silicon nitride (Si_3_N_4_) membrane windows at the Center for Functional Nanomaterials, Brookhaven National Laboratory (BNL). The silicon nitride membrane arrays were provided commercially by Silson. The windows are 500 μm × 500 μm and 200 nm thick in 24 × 24 or 9 × 7 arrays on silicon wafers. Before gold deposition on the membrane, a 2-nm titanium adhesion layer was deposited. To minimize impurities during deposition, the preparation was performed at a pressure of around 10^−6^ mbar. The sample thicknesses reported here are nominal values, estimated from the deposition rate measured on a quartz crystal balance. We observed no diffraction signal contributions from the titanium. For the 300-nm thin film, the grain sizes were estimated to be 163 ± 40 nm using the Scherrer formula, determined in a separate measurement of the (111) crystal peak width at the Advanced Photon Source, sector 34-ID-C.

### Time-resolved x-ray diffraction

We performed TR XRD on the polycrystalline gold films at the PAL-XFEL. The sample was mounted perpendicular to the XFEL beam. The optical pump beam was a few degrees away from normal incidence in an almost colinear geometry. The pump pulse of 400 nm and 100 fs was generated from an 800-nm Ti:sapphire regenerative amplifier laser system (Coherent, Legend), frequency-doubled with a barium borate (BBO) crystal. The choice of 400 nm gives better optical coupling with the polycrystalline gold film than 800 nm since less light is reflected. The laser beam size was estimated by monitoring the laser power transmitted power through an aperture at the sample position. With a 200-μm aperture, 90% of the laser power was transmitted. This means that the 1/*e*^2^ beam size, which is often called the laser beam diameter, is less than 200 μm. From this, we estimate that the FWHM laser beam size, *d*, is around 100 μm, which is the distance between the 50% intensity points. The incident laser fluence (*F*_in_) was then calculated conventionally as pulse energy (*E*_pulse_) divided by the area, *E*_pulse_/*A*, where *A* = π(*d*^2^/4). For our experiment, we used a range of incident pulse energies from 5 to 300 μJ, resulting in an incident laser fluence of 63 mJ/cm^2^ to 3.8 J/cm^2^ at the sample position. To probe the uniformly excited part of the sample, the monochromatic XFEL beam was focused to 25 μm (FWHM) spot size at the sample position with compound refractive lenses. Temporal overlap of both beams was achieved using a GaAs metal-semiconductor-metal detector (Hamamatsu) at the sample position, while the spatial overlap was achieved by centering both beams on a 100-μm-diameter pinhole moved onto the sample position, as seen in Fig. 1A. The sample was mounted on a motorized scanner that allows single-shot measurement. The scanner was synchronized to the XFEL beam for “mesh” scans, visiting each window of the array once for each pump-probe delay time. A diffraction image from each shot was collected with a Rayonix MX225-HS area detector, 2 × 2 binned, at 10 Hz. The direct beam intensity was recorded with a quadrant beam position monitor before the sample and on a photodiode after the sample. To disregard sample damage, only the first shot on each window was considered in the data analysis. The optical taper geometry of the area detector was precalibrated as a fixed correction in the hardware. The data were background-subtracted, using both white-field and dark-field corrections, with the latter remeasured once per day. CeO_2_ powder was used as a calibrant to correct for possible drifts of photon energy and small variations in the sample-to-detector distance between sample changes.

### Data analysis and line shape fitting

As a first step, the calibration diffraction image was fitted using Fit2D to refine the sample-to-detector distance, detector orientation angles, and center pixel position (*38*). Then, all the refined values were transferred to PyFAI for azimuthal integration of the measured diffraction images (*39*). The direct beam was measured with a photodiode after the sample, and the data were normalized to the incident photon flux recorded at each shot. XRD images were collected at different pump-probe delay times. After calibration, normalization, and integration, each crystalline diffraction peak of the pristine sample was fitted with a sum of two Gaussian functions with constrained intensity ratio and constrained to the same peak position. The peak shape is determined by different factors such as the grain size distribution, the detector resolution, and the beam divergence and is often reported to be Voigt-shaped. However, our analysis showed that the two-Gaussian peak gave a better fit to the data. The XRD curves for (111) at positive pump-probe delay time showed two distinct peaks: the (111) crystal peak and the neighboring intermediate peak. In the peak fitting here, the (111) crystal peak shape was taken to be the same fixed-ratio sum of two Gaussian peaks, while the new intermediate peak was modeled with a third single-Gaussian peak. The fitting was performed in Python using the lmfit package, which uses nonlinear least-squares fitting. To limit the fit parameters, the following constraints were introduced:

1) The peak positions of the two Gaussians used for the crystal peak were the same. The widths of the two Gaussian components were fixed at 0.0212 and 0.063 Å^−1^ (FWHM), optimized by fitting the negative pump-probe delay time data where this is the only peak.

2) Similarly, the peak intensity ratio between peaks 1 and 2 was fixed to a value of 2.47 optimized by fitting the negative pump-probe delay time data.

3) The intermediate peak was turned on only at the positive pump-probe delay time. The peak position, width, and amplitude were all allowed to be free during the fit.

This line shape fitting procedure was used to obtain the parameters plotted in Figs. 3 to 5. In addition, we extracted the powder and intermediate peak positions for the different film thicknesses. The intermediate peak position is reported at the time delay, where we observe the maximum change. For the 300-nm film, the powder peaks were at 2.6797, 3.096, and 5.125 Å^−1^ for the (111), (200), and (311), respectively, and the corresponding intermediate peaks were at 2.655, 3.07, and 5.048 Å^−1^. Similarly, for the 100-nm film, (111), (200), and (311) powder peaks were at 2.693, 3.11, and 5.14 Å^−1^, respectively, and we were able to resolve only the intermediate peak close to (111) at 2.648 Å^−1^. However, for the 50-nm thin film, we could only determine the position of (111) and (200) peaks at 2.703 and 3.117 Å^−1^, respectively, and the intermediate peak positions were not resolved. The powder peak positions increase slightly with decreasing thickness, perhaps due to lattice contractions, as reported before (*40*).
